# Semi-automated prediction approach of target shifts using machine learning with anatomical features between planning and pretreatment CT images in prostate radiotherapy

**DOI:** 10.1093/jrr/rrz105

**Published:** 2020-01-29

**Authors:** Yudai Kai, Hidetaka Arimura, Kenta Ninomiya, Tetsuo Saito, Yoshinobu Shimohigashi, Akiko Kuraoka, Masato Maruyama, Ryo Toya, Natsuo Oya

**Affiliations:** 1 Department of Health Sciences, Graduate School of Medical Sciences, Kyushu University, 3-1-1, Maidashi, Higashi-ku Fukuoka 812-8582, Japan; 2 Department of Radiological Technology, Kumamoto University Hospital, 1-1-1, Honjo, Chuo-ku, Kumamoto, 860-8556, Japan; 3 Department of Health Sciences, Faculty of Medical Sciences, Kyushu University, 3-1-1, Maidashi, Higashi-ku Fukuoka 812-8582, Japan; 4 Department of Radiation Oncology, Kumamoto University Hospital, 1-1-1, Honjo, Chuo-ku, Kumamoto, 860-8556, Japan

**Keywords:** prostate radiotherapy, cone beam computed tomography, anatomical features, target shifts, machine learning

## Abstract

The goal of this study was to develop a semi-automated prediction approach of target shifts using machine learning architecture (MLA) with anatomical features for prostate radiotherapy. Our hypothesis was that anatomical features between planning computed tomography (pCT) and pretreatment cone-beam computed tomography (CBCT) images could be used to predict the target, i.e. clinical target volume (CTV) shifts, with small errors. The pCT and daily CBCT images of 20 patients with prostate cancer were selected. The first 10 patients were employed for the development, and the second 10 patients for a validation test. The CTV position errors between the pCT and CBCT images were determined as reference CTV shifts (teacher data) after an automated bone-based registration. The anatomical features associated with rectum, bladder and prostate were calculated from the pCT and CBCT images. The features were fed as the input with the teacher data into five MLAs, i.e. three types of artificial neural networks, support vector regression (SVR) and random forests. Since the CTV shifts along the left–right direction were negligible, the MLAs were developed along the superior–inferior and anterior–posterior directions. The proposed framework was evaluated from the residual errors between the reference and predicted CTV shifts. In the validation test, the mean residual error with its standard deviation was 1.01 ± 1.09 mm in SVR using only one feature (one click), which was associated with positional difference of the upper rectal wall. The results suggested that MLAs with anatomical features could be useful in prediction of CTV shifts for prostate radiotherapy.

## INTRODUCTION

Among men, prostate cancer (PCa) is the second most commonly diagnosed cancer worldwide [1, 2]. External-beam radiation therapy (EBRT) is the treatment selected for about one-third of patients with localized PCa, and this proportion increases with age [3]. Intensity-modulated radiotherapy (IMRT) provides a highly conformal radiation dose distribution while minimizing the toxicity to the surrounding normal organs, and IMRT has become the most common form of EBRT delivery for PCa [4]. However, the target (prostate and/or seminal vesicles) positions during treatment (fractions) may change with variations in the positions, volumes and/or shapes of the rectum and bladder [5, 6]. In prostate IMRT, therefore, patient positioning has been performed on image guided radiotherapy (IGRT) systems based on the image registration between the planning computed tomography (pCT) and pretreatment cone-beam computed tomography (CBCT) images in order to correct for target position variations [4, 7–12]. In general, many institutions use the following two-step image guided procedure for patient positioning after manual positioning based on skin markers with a laser: (i) automated bone-based patient positioning and then (ii) manual and subjective target-based fine positioning, in which the target shifts are determined based on clinical expertise [10, 13, 14]. However, the second step of target-based positioning could cause inter- and intra-observer variations in CBCT images [11, 12]. Consequently, the target positioning observer variations could decrease local tumor control and increase normal tissue toxicity, because IMRT can produce highly conformal dose distributions with rapid dose fall-off. If patient positioning was automatically performed using a computer, by a one-step procedure based on the target, results could be obtained with reproducibility and efficiency.

Yoshidome *et al*. proposed an automated framework for estimating lung tumor locations for tumor-based patient positioning with megavoltage (MV)-CBCT during stereotactic body radiotherapy [14]. They used a tumor-template matching technique with an enhancement of the tumor region adopting image processing filters, and their framework could automatically predict lung tumor locations with errors of <1 mm for a lung screening phantom and 2 mm for clinical cases. However the prostate boundary is difficult to recognize in CBCT images [15], so adaptation of the template-matching technique for prostate cases would be challenging.

Recently, experience- and knowledge-based approaches using machine learning architectures (MLAs) have been applied in prediction of prostate location [16, 17]. Zhao *et al*. proposed a deep learning (DL) model for localization of prostate based on projection kilo-voltage X-ray images acquired prior to or during radiation therapy [16]. Their study was the first attempt at applying DL to IGRT. Their DL model could estimate the prostate locations to within <1.7 mm on average. However, their framework was designed to predict the prostate locations from 2D projection images, and not for CBCT-based IGRT. Ninomiya *et al*. attempted to predict the prostate locations using MLAs, which were an artificial neural network (ANN), a support vector machine (SVM) and random forests (RF), with anatomical feature points in Bayesian delineation frameworks [17]. They selected the lowest point of the bladder, the contact point of the bladder with the prostate, the center of the prostate, the front point of the rectum and the rear point of the rectum as the anatomical feature points on CT images. The prostate locations were predicted using MLAs that learned the relationships between the reference prostate locations and feature points, thus their framework could predict the prostate locations with 1.8 mm on average for the SVM. However, the approach considered only prostate location for the purpose of delineation of the prostate. Furthermore, their anatomical feature points represented only the positional information of rectum, bladder and prostate. The shifts of prostate and seminal vesicles were reportedly associated with bladder and/or rectal volume change as well as with positional information [5, 6]. Therefore, anatomical features associated with position and/or volume change of rectum, bladder and prostate between pCT and pretreatment CBCT images could be useful for prediction of daily target shifts, i.e. clinical target volume (CTV) shifts.

The goal of this study was to develop a semi-automated prediction approach for CTV shifts using MLAs with anatomical features between pCT and pretreatment CBCT images for PCa patients’ positioning in IGRT. Our hypothesis was that anatomical features correlated with the CTV shifts and could be used to predict the CTV shifts with small errors.

## MATERIALS AND METHODS

This study was approved by the institutional review board of our university hospital. The CTV position errors between the pCT and daily pretreatment CBCT images were determined based on clinical expertise after an automated bone-based registration, and they were employed as the reference CTV shifts. Anatomical features associated with the rectum, bladder and prostate were selected from the pCT and pretreatment CBCT images. The features and corresponding reference CTV shifts were used as the input and teacher data, respectively, for five MLAs, i.e. the three types of ANNs, a regression model based on the SVM (support vector regression, SVR) and RF. Finally, the CTV shifts were predicted from the anatomical features using the developed MLAs. The proposed framework was evaluated using the residual errors between the reference and predicted CTV shifts.

### Clinical cases

Twenty consecutive patients (median age: 76.6 years; range: 55–85 years; stage: T1c–T3b, N0 and M0; and risk groups: 8 intermediate and 12 high-risk patients) with PCa without fiducial marker, who underwent image-guided IMRT, were selected with pCT and daily pretreatment CBCT images in our university hospital from January 2014 to March 2017 for this study. A total dose of 76 Gy was delivered to the planning target volume (PTV) of each patient at 38 fractions using a linear accelerator (Synergy™, Elekta Oncology Systems, Crawley, West Sussex, UK). CTV was the prostate with the seminal vesicles, and a CTV-to-PTV margin of 7 mm was applied except the posterior margin of 4 mm. The 20 patients were randomly divided into two datasets: training datasets including the first 10 patients with the pCT and pretreatment CBCT images at 378 fractions, and validation datasets including the second 10 patients with the pCT and pretreatment CBCT images at 100 fractions. In the validation datasets, the images at 10 fractions were selected per patient. The training datasets were employed for the development of the MLAs based on a leave-one-out-by-patient cross-validation (LCV) test, and the validation datasets were used for the validation test of the proposed framework.

A CT scanner (LightSpeed RT; GE, Amersham, UK) was employed to acquire the pCT images with dimensions of 512 × 512 pixels, in-plane pixel size of 1.27 mm and slice thickness of 2.5 mm. In the pCT scanning and treatment sessions, the patients were immobilized with a vacuum bag, thermoplastic body shell and carbon base plate (ESN-1800; Engineering System, Nagano, Japan) in the supine position. The CBCT images with the dimensions of 512 × 512 pixels, in-plane pixel size of 0.801 mm and slice thickness of 1.0 mm were acquired on an IGRT system (XVI™ version 4.5, Elekta Oncology Systems, Crawley, West Sussex, UK) at each treatment session for the patients, prior to irradiation for correction of the CTV locations. However, the pretreatment CBCT acquisitions in 2 fractions of the training datasets were not performed because of device failures. All patients were asked to defecate, and their bladders were filled with ~300 mL of urine.

**Fig. 1.**
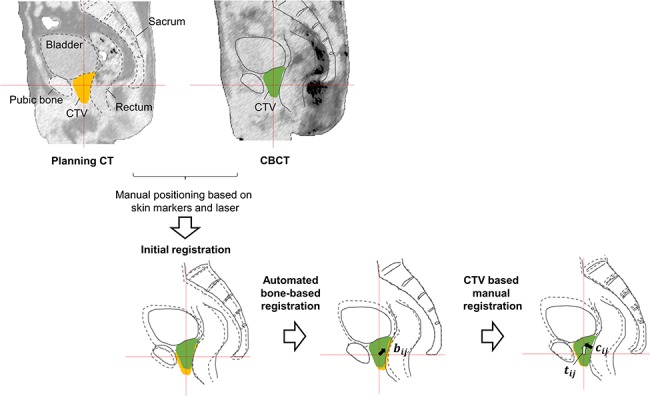
Workflow of patient positioning with image guidance based on CT and pretreatment CBCT for prostate intensity-modulated radiotherapy in sagittal view. The shift after the automated bone-based registration was ${\boldsymbol{b}}_{ij}$, and that after the CTV based manual registration was ${\boldsymbol{c}}_{ij}$. The ${\boldsymbol{c}}_{ij}$ was employed as the reference CTV shift. Total patient shift is indicated as ${\boldsymbol{t}}_{ij}.$

### Acquisition of CTV shifts

**Fig. 2.**
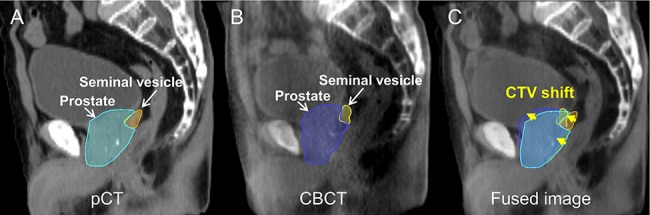
Illustration of the CTV shift of the prostate and seminal vesicle: (**A**) prostate and seminal vesicle rendered in light blue and orange in the pCT image, respectively, (**B**) prostate and seminal vesicle rendered in blue and yellow in a CBCT image, respectively, and (**C**) CTV shift indicated by the yellow arrows in the pCT/CBCT fusion image after an automated bone-based registration.

The workflow of patient positioning with image guidance is shown in Fig. 1. The patient positioning was performed using a two-step image-guided procedure after manual positioning based on skin markers with a laser, using the first automated bone-based registration between the pCT and pretreatment CBCT images followed by the second manual and subjective CTV-based fine positioning. The shifts in the first step ${\boldsymbol{b}}_{ij}$ and the second step ${\boldsymbol{c}}_{ij}$ were retrospectively obtained along the left–right (LR), superior–inferior (SI) and anterior–posterior (AP) translational planes for the *i-*th treatment fraction of the *j*-th patient from the clinical patient position correction data. Positive values for LR, SI and AP indicated the left, superior and anterior CTV shifts, respectively. Total patient shift was indicated as ${\boldsymbol{t}}_{ij}.$ The ${\boldsymbol{c}}_{ij}$, which was determined with the consensus of two radiation technologists and a radiation oncologist, was employed as a reference CTV shift. Figure 2 shows an example of the CTV shift on the sagittal plane. The mean absolute value and standard deviation (SD) of the CTV shifts along each direction were calculated from the CTV shifts of 20 patients. Since the CTV shifts along the LR direction were negligible (mean absolute value <0.1 mm, SD <0.5 mm), the MLAs were developed for prediction of the CTV shifts along the SI and AP directions. Additionally, the agreement between the reference CTV shifts and the CTV shifts determined independently by a medical physicist, who was not involved in the determination of the reference CTV shifts, was evaluated based on the Cohen’s kappa coefficient [18] and residual errors using validation datasets to investigate the reproducibility of the reference data.

**Table 1:** Summary of the nine anatomical features and the definitions of the distances for determination of rectum and bladder anatomical features 1–7 (Fig. 3). Feature points S and I are defined as the superior–posterior and inferior–posterior edge of the pubic bone in the sagittal plane at the isocenter level

No.	Definition
**Anatomical features**	
1	Positional difference of the posterior-wall of bladder
2	Positional difference of the superior-wall of bladder
3	Positional difference of the upper rectal wall
4	Difference of the upper rectal diameter
5	Positional difference of the lower rectal wall
6	Difference of the lower rectal diameter
7	Difference of the rectal shape
8	Positional differences of the center points of the prostate along the SI direction
9	Positional differences of the center points of the prostate along the AP direction
**Distances for determination of the anatomical features 1–7**	
1	Distance between feature point S and top of bladder posterior convex
2	Distance between feature point S and the intersection point of a perpendicular line in the SI axis from feature point S and bladder superior-wall
3	Distance between feature point S and the intersection point of a perpendicular line in the AP axis from feature point S and rectal anterior-wall
4	Diameter of the rectum in the same AP axis as distance 3
5	Distance between feature point I and the intersection point of a perpendicular line in the AP axis from feature point I and rectal anterior-wall
6	Diameter of the rectum in the same AP axis as distance 5
7	Difference of distances 4 and 6 (distance 6 – distance 4)

### Anatomical features

The movements of the bladder and upper rectum and the volume changes of the bladder were associated with the shifts of prostate and seminal vesicles, and the volume changes of upper and lower rectum were associated with the seminal vesicles shifts [6]. Furthermore, the prostate and seminal vesicles reportedly shifted along a diagonal direction extending from an anterior–superior position to a posterior–inferior position in the sagittal plane [6]. Therefore, the anatomical features of rectum, bladder and prostate extracted from the sagittal plane were assumed to correlate with the CTV shifts and be useful for prediction of the CTV shifts along the SI and AP directions in this study.

**Fig. 3.**
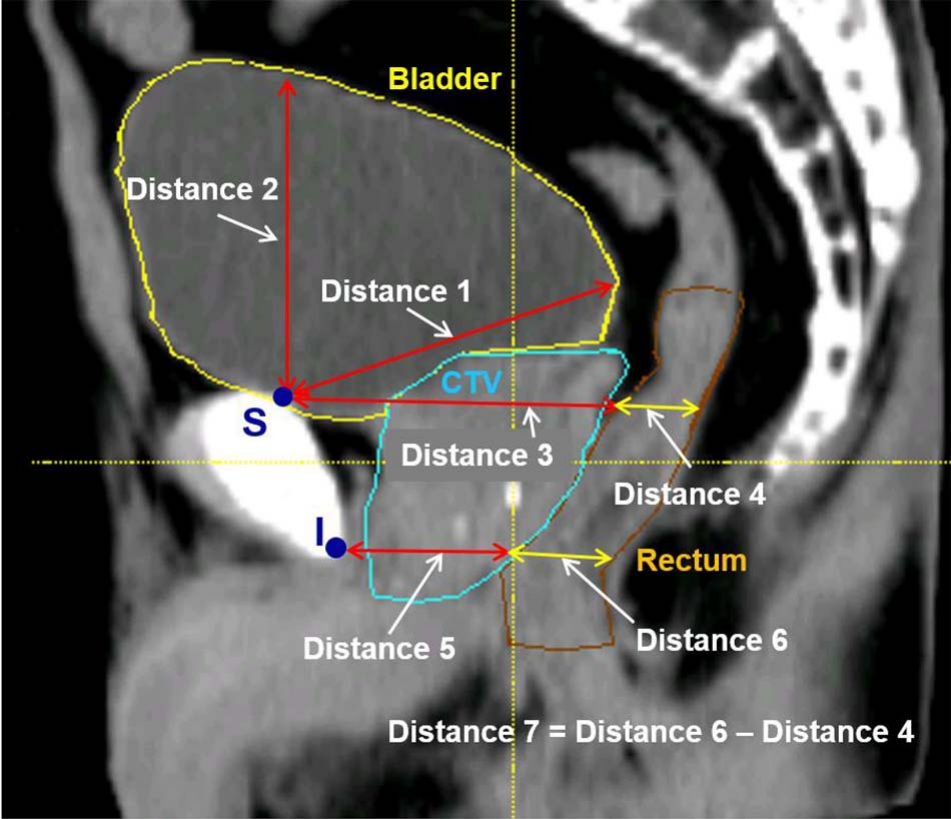
Definitions of distances 1–7 to determine the rectum and bladder anatomical features (features 1–7) selected from a planning CT image. The superior–posterior edge of the pubic bone was defined as feature point S, and the inferior–posterior edge of the pubic bone was defined as feature point I. The features express the variations of the bladder and rectum based on the pelvic bone.

A total of nine anatomical features were extracted from the sagittal plane at the isocenter levels of the pCT and pretreatment CBCT images after an automated bone-based registration in the treatment fractions. The nine anatomical features are summarized in Table 1. Among them, seven anatomical features were obtained by manual measurements of the seven geometrical distances in the pCT and pretreatment CBCT images that could be associated with the variations of the position and volume of the rectum and bladder. Distances 1–7 are listed in Table 1 and shown in Fig. 3. The features were calculated as the differences of distances 1–7 between the pCT and pretreatment CBCT images as follows:

(1) \begin{equation*}\;\;\;\;\;\;\;\;\;\;\;\;\;\;\;\;\;\;\; {f}_l={d}_l^{CBCT}-{d}_l^{pCT}\left(l=1\;\mathrm{to}\;7\right), \end{equation*}

where ${f}_l$ was the *l*-th feature$.$ The parameters ${d^{CBCT}}_l$ and ${d^{pCT}}_l$ were the *l*-th distances in the pretreatment CBCT and pCT images. Feature point S was defined as the superior–posterior edge of the pubic bone on the sagittal plane, and feature point I was defined as an inferior–posterior edge of the pubic bone, as shown in Fig. 3. Features 1 and 2 were related to the variations of the bladder volume, and features 3, 4, 5 and 6 were related to the variations in the upper and lower rectal position and volume and the contraction of the levator ani muscle. Feature 7 could reflect the variation of the rectal shape. Additionally, features 8 and 9 were obtained as the positional differences of the center points of the prostate (COP) along the SI and AP directions between pCT and pretreatment CBCT images. The COP were selected manually as the middle points of the prostate along the SI and AP directions in the pCT and pretreatment CBCT images. The relationships between each anatomical feature and the CTV shifts were analysed based on Spearman’s rank-correlation test, and the anatomical features with a higher correlation to the CTV shifts were identified using their correlation coefficients. All statistical analyses in this study were carried out using JMP® 14 (SAS Institute Inc., Cary, NC, USA).

### Machine learning architectures

The CTV shifts were predicted using five types of MLAs: ANN with Bayesian regularization backpropagation (BR-ANN), Levenberg-Marquardt backpropagation (LM-ANN), scaled conjugate gradient backpropagation (SCG-ANN), SVR and RF. The MLAs were implemented using the MATLAB Neural Network Toolbox and Statistics and Machine Learning Toolbox (MathWorks, Inc., Natick, MA).

### Artificial neural network

The ANN is a popular statistical method that can learn the relationships between the input features and teaching data [19]. In general, some backpropagation algorithms are adopted to optimize the ANNs, e.g. BR, LM and SCG [19–21]. In this study, BR was implemented within the LM optimization, and adjustment parameter *μ* was set to 0.005 and 0.001 as the initial values in the BR-ANN and LM-ANN, and the scalar parameter λ was set to $5.0\times{10}^{-7}$ in the SCG-ANN.

### Support vector regression

The SVM can classify data into several (generally two) categories based on the output of a discriminant function. The SVM constructs a discriminant function in a linearly separable space by applying a non-linear kernel function to a given training dataset [22]. SVR is a regression method based on SVM and the structured risk minimization principle. In this study, the Gaussian kernel (radial bases function kernel) was used as a kernel function, and a regularization parameter C was set [interquartile range of ${\boldsymbol{c}}_{ij}$ used leaning/1.349] as the default value in the MATLAB Statistics and Machine Learning Toolbox.

### Random forests

The RF method [23] is a combination of multiple decision trees, and each tree is constructed by a random vector sampled from the training dataset. The decision tree consists of a node and link, and the first node is referred to as the root node. The decision tree reaches the terminal node using the bootstrap sample as the training data while splitting each node from a random selection of the features. In this study, the minimum number of data per leaf node to stop the growing tree was set to 5.

**Fig. 4.**
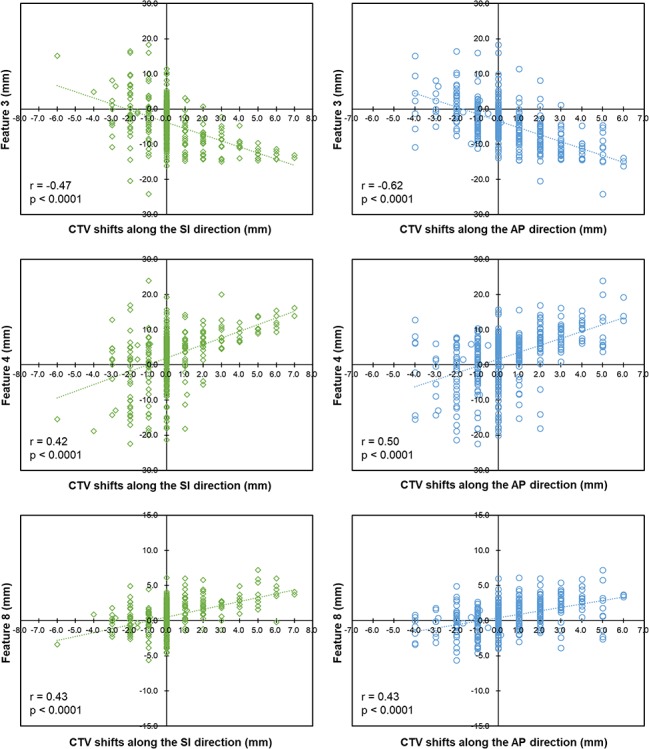
Correlation coefficients (*r*) of the three highest correlated features with the CTV shifts along the SI and AP directions.

### Leave-one-out-by-patient cross-validation test

The LCV test was employed for the training and evaluation for the MLAs developed using the training datasets. In this test, one patient was left out from the training datasets as the test patient, and the regression models were developed using the remaining patient data. MLAs were developed for the SI and AP directions, respectively, which were composed of anatomical features (input data) and corresponding reference CTV shifts (teacher data). The anatomical features were fed into the regression models, and the CTV shifts were predicted for testing the models. The anatomical features were extracted by a medical physicist with 10 years of experience for 5 patients and a radiotherapy technologist with 5 years of experience for another 5 patients because MLAs can be generalized with the anatomical features extracted by different professionals. The residual errors between the reference and predicted CTV shifts were obtained to evaluate each MLA along the SI and AP directions, respectively. The optimal ML parameters were investigated by changing each ML parameter one by one, i.e. the number of nodes in a hidden layer from 4 to 10 in ANN, the ε value (half the width of the ε-insensitive band) of 0.01, 0.05, 0.1, 0.3, 0.5 and 1.0 in SVR, and the number of decision trees of 10, 30, 50, 70, 100 and 150 in RF, so that the smallest training errors between the reference and predicted CTV shifts could be achieved. The optimal number of features was searched by adding the features one by one (1–9) in order of higher correlation with CTV shifts.

### Validation test

A validation test for evaluation of the proposed framework was carried out using the validation datasets. The anatomical features, which were extracted by another medical physicist with >10 years of experience from the pCT and pretreatment CBCT images at 100 fractions of the validation datasets, were fed into the five MLAs developed by the training datasets, and the CTV shifts were predicted. The optimal ML parameters for each MLA in the LCV test were used in this validation test. The results of MLAs were compared with that of a conventional soft tissue matching method, i.e. a grey value match based on a correlation ratio algorithm on the XVI system [24]. The CTV shifts were estimated by using the grey value match after the automated bone-based registration with setting the region of interest as CTV plus 5 mm margin [24] for 478 CBCT images (total 478 fractions).

## RESULTS

### Analysis of the reference CTV shifts

The mean absolute values ± the SDs of the reference CTV shifts were 0.9 ± 1.4 mm along the SI direction and 1.3 ± 1.3 mm along the AP direction, respectively. The Cohen’s kappa coefficients were 0.73 (*P* < 0.0001) along the SI direction and 0.53 (*P* < 0.0001) along the AP direction, which denoted substantial and moderate agreement [18], respectively. The mean absolute residual errors were 0.3 ± 0.5 mm along the SI direction and 0.6 ± 0.7 mm along the AP direction. The percentages of inter-observer agreement within 2.0 mm were 99 and 100% along the SI and AP directions, respectively. Since the CTV shifts were reproducible, they were used as reference CTV shifts.

### Correlation coefficients between each anatomical feature and the reference CTV shifts

Figure 4 shows the correlations of the three highest correlated features with the reference CTV shifts along the SI and AP directions. The correlation coefficients between the anatomical features and CTV shifts are listed in Table 2. The anatomical features were written in order from the highest to lowest correlation with the CTV shifts. Feature 3 (positional difference of the upper rectal wall) had the highest correlation with the CTV shifts with correlation coefficients of −0.47 (*P* < 0.0001) and −0.62 (*P* < 0.0001) along the SI and AP directions, respectively. Feature 2 (positional difference of the superior-wall of the bladder) had the lowest correlation with the CTV shifts with correlation coefficients of −0.09 (*P* = 0.0976) and −0.19 (*P* = 0.0002) along the SI and AP directions, respectively. From the results of the correlation coefficients, the number of features and the corresponding features used were determined as shown in Table 3. Further, the numbers of clicks on the CBCT images to acquire the features are also shown.

**Table 2:** Correlation coefficients with the *P*-values between each anatomical feature and the CTV shifts. The features are listed in the highest to lowest order of correlation with the CTV shifts. Asterisk (*) indicates *P* < 0.0001

	Correlation coefficients with CTV shifts		
			Mean absolute value
Feature number	SI	AP	of SI and AP
3	−0.47^*^	−0.62^*^	0.55
4	0.42^*^	0.50^*^	0.46
8	0.43^*^	0.43^*^	0.43
9	0.26^*^	0.51^*^	0.38
1	−0.21^*^	−0.40^*^	0.31
6	0.36^*^	0.20^*^	0.28
7	−0.16 (*P* = 0.0015)	−0.35^*^	0.26
5	−0.23^*^	−0.15 (*P* = 0.0026)	0.19
2	−0.09 (p = 0.0976)	−0.19 (*P* = 0.0002)	0.14

**Table 3:** The number of features and the corresponding features used with the number of clicks on CBCT images needed to acquire the features. The features used were determined by adding the features one by one in the order of higher correlation with CTV shifts

Number of features	Features used	Number of clicks
1	Feature 3	1
2	Features 3, 4	2
3	Features 3, 4, 8	3
4	Features 3, 4, 8, 9	3
5	Features 3, 4, 8, 9, 1	4
6	Features 3, 4, 8, 9, 1, 6	5
7	Features 3, 4, 8, 9, 1, 6, 7	5
8	Features 3, 4, 8, 9, 1, 6, 7, 5	6
9	All features	7

### Leave-one-out-by-patient cross-validation test

The residual errors for the LCV test are summarized with various numbers (1–9) of the anatomical features in Table 4. The mean absolute residual errors with SDs were 1.02 ± 0.99 (BR-ANN), 1.04 ± 0.99 (LM-ANN), 1.04 ± 0.93 (SCG-ANN), 1.01 ± 1.07 (SVR) and 1.02 ± 0.97 (RF) mm with the optimal number of features. However, there were no significant differences in the residual errors among various numbers of features for each MLA (*P* > 0.05, Steel-Dwass test).

**Table 4:** Mean absolute residual errors with standard deviations (mm) for various numbers of features using a leave-one-out-by-patient cross-validation test for the training datasets. Asterisk (^*^) indicates the smallest mean error in each machine learning architecture

	Number of features								
	1	2	3	4	5	6	7	8	9
**BR-ANN**									
SI	1.12 ± 1.30	1.19 ± 1.29	1.05 ± 1.02	1.05 ± 1.10	1.13 ± 1.05	1.10 ± 1.08	1.15 ± 1.16	1.19 ± 1.10	1.13 ± 1.08
AP	1.20 ± 1.02	1.20 ± 1.00	1.13 ± 0.99	1.00 ± 0.87	1.01 ± 0.87	1.03 ± 0.87	1.03 ± 0.86	1.05 ± 0.89	1.07 ± 0.90
Mean	1.16 ± 1.17	1.20 ± 1.16	1.09 ± 1.00	1.02 ± 0.99^*^	1.07 ± 0.96	1.07 ± 0.98	1.09 ± 1.02	1.12 ± 1.01	1.10 ± 1.00
**LM-ANN**									
SI	1.10 ± 1.28	1.12 ± 1.27	1.05 ± 1.07	1.09 ± 1.21	1.12 ± 1.09	1.01 ± 0.91	1.01 ± 1.06	1.09 ± 1.30	1.02 ± 1.06
AP	1.21 ± 1.11	1.22 ± 1.01	1.18 ± 1.02	1.05 ± 0.88	1.04 ± 0.89	1.13 ± 0.95	1.06 ± 0.91	1.05 ± 0.91	1.08 ± 0.88
Mean	1.16 ± 1.20	1.17 ± 1.15	1.11 ± 1.05	1.07 ± 1.06	1.08 ± 1.00	1.07 ± 0.93	1.04 ± 0.99^*^	1.07 ± 0.98	1.05 ± 0.97
**SCG-ANN**									
SI	1.13 ± 1.29	1.09 ± 1.24	1.08 ± 1.18	1.13 ± 1.05	1.12 ± 1.10	1.11 ± 1.00	1.07 ± 1.03	1.04 ± 0.96	1.11 ± 1.22
AP	1.17 ± 1.00	1.16 ± 1.02	1.13 ± 0.99	0.99 ± 0.86	1.02 ± 0.91	1.02 ± 0.90	1.06 ± 0.91	1.04 ± 0.90	1.03 ± 0.87
Mean	1.15 ± 1.15	1.13 ± 1.14	1.11 ± 1.09	1.06 ± 0.96	1.07 ± 1.01	1.06 ± 0.95	1.07 ± 0.97	1.04 ± 0.93^*^	1.07 ± 1.06
**SVR**									
SI	0.97 ± 1.36	1.03 ± 1.44	0.92 ± 1.22	0.96 ± 1.21	0.99 ± 1.23	0.96 ± 1.21	0.97 ± 1.22	1.02 ± 1.27	1.02 ± 1.28
AP	1.23 ± 1.10	1.17 ± 1.05	1.12 ± 0.97	1.06 ± 0.89	1.04 ± 0.91	1.08 ± 0.93	1.09 ± 0.94	1.09 ± 0.94	1.10 ± 0.93
Mean	1.10 ± 1.24	1.10 ± 1.26	1.02 ± 1.11	1.01 ± 1.07^*^	1.02 ± 1.08	1.02 ± 1.08	1.03 ± 1.09	1.05 ± 1.12	1.06 ± 1.12
**RF**									
SI	1.21 ± 1.34	1.19 ± 1.33	1.06 ± 1.13	1.06 ± 1.08	1.07 ± 1.09	1.01 ± 1.06	1.01 ± 1.05	1.00 ± 1.04	1.01 ± 1.07
AP	1.31 ± 1.05	1.25 ± 1.01	1.13 ± 0.96	1.04 ± 0.88	1.03 ± 0.88	1.03 ± 0.88	1.06 ± 0.90	1.06 ± 0.91	1.05 ± 0.90
Mean	1.26 ± 1.21	1.22 ± 1.18	1.09 ± 1.05	1.05 ± 0.99	1.05 ± 0.99	1.02 ± 0.97^*^	1.04 ± 0.98	1.03 ± 0.97	1.03 ± 0.99

**Table 5:** Mean absolute residual errors with standard deviations (mm) of our proposed methods (by using only one feature) and the grey value match by an IGRT system (XVI) using validation datasets.

	Machine learning architectures					
						IGRT system (XVI)
	BR-ANN	LM-ANN	SCG-ANN	SVR	RF	grey value match
SI	1.03 ± 0.88 (0.00–4.73)	1.07 ± 1.04 (0.00–4.38)	1.02 ± 0.83 (0.01–4.65)	0.81 ± 1.00 (0.00–4.65)	1.00 ± 0.98 (0.01–4.55)	2.1 ± 2.9 (0.00–25.5)
AP	1.27 ± 1.03 (0.01–6.63)	1.17 ± 1.09 (0.03–5.24)	1.16 ± 0.99 (0.03–6.15)	1.22 ± 1.15 (0.00–6.01)	1.30 ± 1.09 (0.00–6.33)	1.9 ± 2.2 (0.00–15.4)
Mean	1.15 ± 0.97	1.12 ± 1.07	1.09 ± 0.92	1.01 ± 1.09	1.15 ± 1.05	2.0 ± 2.6

### Validation test

**Fig. 5.**
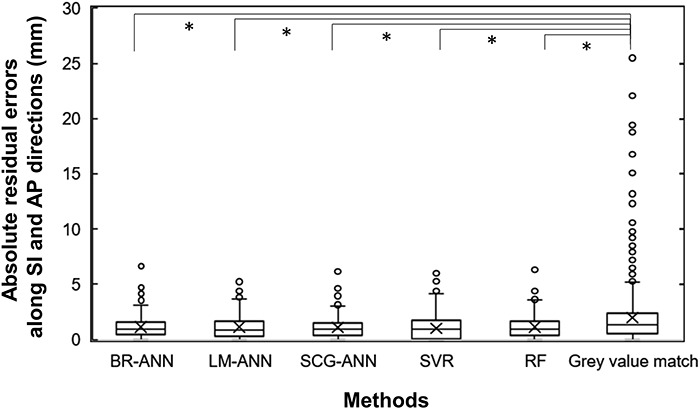
Box plots of the residual errors for MLAs and grey value match. Asterisks indicate the statistically significant differences (*P* < 0.05, Steel-Dwass test).

**Fig. 6.**
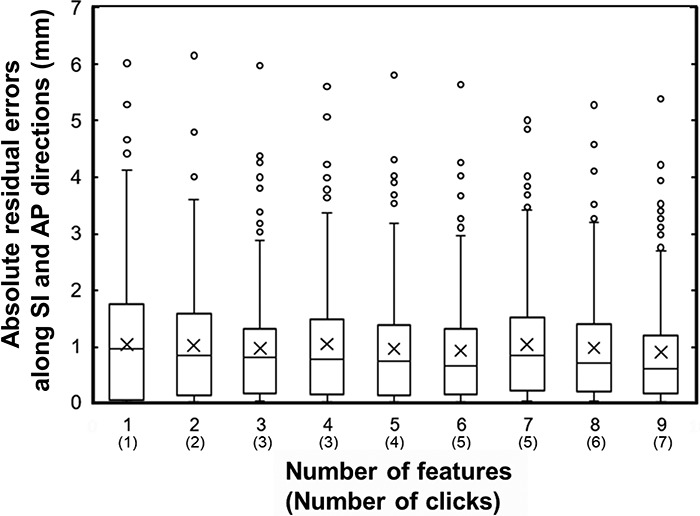
Relationship between absolute residual errors along the SI and AP directions and the number of features in SVR for the validation test. The number of clicks to acquire the features are shown in parentheses.

In the LCV test, there were no significant differences in the residual errors among various numbers of features for each MLA, thus, the validation test was carried out using only one feature (feature 3, positional difference of the upper rectal wall). The mean absolute residual errors with SDs for the validation test are summarized in Table 5. The residual errors with SDs were 1.15 ± 0.97 (BR-ANN), 1.12 ± 1.07 (LM-ANN), 1.09 ± 0.92 (SCG-ANN), 1.01 ± 1.09 (SVR) and 1.15 ± 1.05 (RF) mm. The maximum errors were 6.63 (BR-ANN), 5.24 (LM-ANN), 6.15 (SCG-ANN), 6.01 (SVR) and 6.33 (RF) mm. There were no significant differences in the residual errors among the MLAs (*P* > 0.05, Steel-Dwass test). The mean residual error with SD in the grey value match was 2.0 ± 2.6 mm with a maximum error of 25.5 mm. There were significant differences in the means of the residual errors between the MLAs and grey value match method (*P* < 0.05, Steel- Dwass test) and the variances of the residual errors between them (*P* < 0.0001, F-test). F-test is a stastical test for equality of variances, which is based on the null hypothesis that two populations have the same variance. The box plots of the residual errors for MLAs and grey value match are shown in Fig. 5, which shows larger errors for the grey value match than for MLAs. In SVR with the smallest errors among the MLAs, the validation tests were carried out using various numbers of features to validate the relationship between prediction errors and the numbers of features. The box plots of the residual errors and the number of features are shown in Fig. 6, and the mean errors ranged from 0.88 (number of features: 9) to 1.02 mm (number of features: 4). There were no significant differences among various numbers of features (*P* > 0.05, Steel-Dwass test).

## DISCUSSION

In this study, an approach for a semi-automated prediction of CTV shifts between pCT and pretreatment CBCT images has been proposed using MLAs with anatomical features for improvement of PCa patients’ positioning. Yoshidome *et al*. showed the usefulness of a tumor-template matching technique on MV-CBCT images for estimating lung tumor locations [14]. On the other hand, the novelty of our study is that the CTV shifts were predicted with small residual errors (<1.3 mm) by using only one anatomical feature (one click) without using the template-matching technique. In clinical practices, the proposed method could be installed in the IGRT system, so that CTV shifts will be obtained by clicking an anatomical feature with a mouse on a sagittal image of CBCT after bone-based registration with pCT images.

**Fig. 7.**
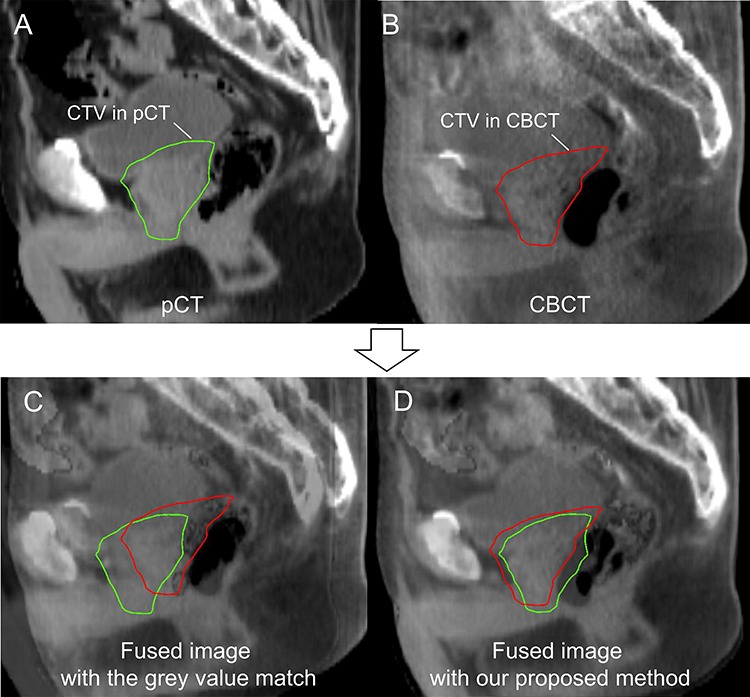
A typical case in which our method had higher prediction accuracy than the grey value match. (**A**) A pCT image with the CTV contour, (**B**) a CBCT image with the CTV contour, (**C**) a fused image with the grey value match, and (**D**) a fused image with SVR using one feature.

The mean absolute residual errors were ≤1.04 mm in all MLAs with each optimal number of features, which minimized the residual errors, in the LCV test. The smallest mean error with its SD of 1.01 ± 1.07 mm was achieved by using SVR with four features. However, there were no significant differences in the residual errors among various numbers of features for each MLA. The validation test was carried out using one feature (feature 3), and the smallest mean error of 1.01 ± 1.09 mm was achieved using SVR. In the validation test of SVR, the mean error was smallest for nine features (Fig. 6), however, there were no significant differences among various numbers of features. Therefore, the prediction of CTV shifts based on only one feature could be feasible, and the operation required for feature selection can be performed simply with only one click (Table 3). In the study by Ninomiya *et al*. [17], the smallest mean prediction error was 1.8 ± 1.0 mm in SVM. They used three anatomical feature points, i.e. the contact point of the bladder and the front point of the rectum, and the center point of the prostate, which should take three clicks on CT images. MLAs produced lower residual errors than the grey value match (*P* < 0.05, Steel- Dwass test) with smaller variabilities than the grey value match (*P* < 0.0001, F-test), i.e. 1.15 ± 0.97 (BR-ANN), 1.12 ± 1.07 (LM-ANN), 1.09 ± 0.92 (SCG-ANN), 1.01 ± 1.09 (SVR), 1.15 ± 1.05 (RF) and 2.0 ± 2.6 mm (grey value match). Figure 7 shows a typical case in which our method (SVR using one feature) had higher prediction accuracy than the grey value match. In that case, although the prediction error was relatively large even in SVR, the grey value match showed a larger error due to the influence of the rectal gas. Barber *et al*. investigated the image registration uncertainty of the grey value match using a male pelvis phantom, and the residual translation errors were <2 mm [24]. On the other hand, in the study by Shi *et al*., the maximum residual error was 16 mm for clinical CBCT images of PCa patients [15]. Residual errors of >10 mm occurred also in our study (Fig. 5), which resemble the results of Shi *et al*. There were small residual errors in the fractions without rectal gas, but there were large errors in the fractions with rectal gas for the grey value match (Figs 5 and 7 and Table 5). More stable results can be obtained with our proposed methods for clinical cases.

**Fig. 8.**
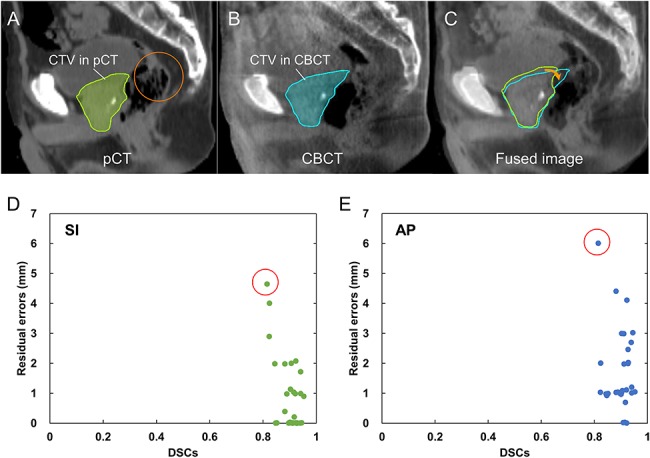
A CTV shift between the pCT and CBCT images at a treatment fraction when a maximum residual error occurred, and the relationships between residual errors and the DSCs along the SI and AP directions. (**A**) a pCT image with the CTV contour, (**B**) a CBCT image with the CTV contour, (**C**) a fused image with contours of CTVs delineated on pCT and CBCT images, (**D**) and (**E**) relationships between residual errors and the DSCs along the SI and AP directions. The stool and gas that existed in pCT (orange circle) disappeared in CBCT images in (B). The red circles in (D) and (E) indicate the maximum errors.

From the results of the correlation coefficients between each feature and the reference CTV shifts, feature 3 (positional difference of the upper rectal wall) had the highest correlation with the CTV shifts (Table 2). In many treatment fractions of the selected patient data, the position and/or volume changes of the upper rectum affected the CTV position. This fact agreed with the results of Zellars *et al*. [6]. Zellars *et al*. found that the prostate and seminal vesicles shifted along a diagonal direction extending from an anterior–superior position to a posterior–inferior position in the sagittal plane, and a statistically significant association was seen in the shifts between the targets (the prostate and seminal vesicles) and upper rectum [6]. Therefore, feature 3 was useful for prediction of the CTV shifts along the SI and AP directions. On the other hand, the features related to the bladder had relatively lower correlation with the CTV shifts than those related to the rectum (Table 2). In particular, feature 2, which reflected the positional difference of the superior-wall of the bladder, had the lowest correlation with the CTV shifts. Reddy *et al*. reported that the bladder expanded anteriorly and superiorly owing to an increase in the volume without pushing the prostate inferiorly [25]. In addition, from our clinical studies, the variations of bladder expansion to the posterior influenced the CTV (the seminal vesicles in particular) shift; however, the variations of bladder expansion to the superior had minimal influence. Therefore, feature 1, which expressed the variation of bladder expansion to the posterior, showed a moderate correlation with the CTV shifts; however, feature 2 showed a low correlation. Features 8 and 9, which represented the positional differences of the COP, were not so highly correlated with CTV shifts. It is assumed that this is because the reference CTV shifts were decided by considering not only the prostate but also the seminal vesicles. 

Delineating the organs on the CBCT images in image guidance for each treatment is time consuming and difficult for low-contrast images. Therefore, in this study, the anatomical features were extracted without delineation of the organs. Recently, an attempt was made by Liu *et al*. [26] to automatically segment the prostate on CT images using a deep neural network (DNN). Their DNN, which trained the 771 prostate glands delineated by one physician, was able to predict the prostate location with 1.1 mm on average. In our proposed method, the prediction accuracy was not inferior to Liu *et al*.’s DNN. Our method could be a simple and effective method because only one click is required for feature selection.

Since the rotation and deformation of the CTVs were not taken into account in this study, their impacts on residual errors were investigated in the relationships between residual errors in SVR (using one feature) and Dice’s similarity coefficients (DSC) of CTVs. Contours of CTVs were delineated on pCT and CBCT images after registration based on reference CTV shifts. The residual errors and DSCs were obtained from the pCT and CBCT images at three treatment fractions of the 10 patients of the validation datasets. Figure 8A, B and C illustrate a CTV shift between the pCT and CBCT images, and Fig. 8D and E depict the relationships between residual errors and the DSCs. There were no significant correlations (correlation coefficients of −0.31 and 0.16 along the SI and AP directions, *P* > 0.05, Spearman’s rank-correlation test) (Fig. 8D and E). The CTV shift between the pCT and CBCT images, at a treatment fraction when a maximum residual error occurred, is shown in Fig 8A, B and C. The maximum errors along the SI and AP directions occurred in the same treatment fraction. The CTV, especially for seminal vesicles, may have been rotated and/or deformed, because the stool and gas that existed in the pCT image disappeared in the CBCT image. The anatomical features in this study may not accurately reflect the rotation and deformation of the CTV.

Whether the manual target positioning is accurate depends on the experience and skills of radiation technologists. Therefore, less-experienced radiation technologists may cause target positioning variations, which could decrease local tumor control and increase normal tissue toxicity. In this study, the reference CTV shifts were manually determined with the consensus of two radiation technologists and a radiation oncologist. As a result, the percentages of inter-observer agreement within 2.0 mm were 99 and 100% along the SI and AP directions, respectively, which were very close to the 99 and 98% along the SI and AP directions, respectively, obtained with fiducial markers [27]. Therefore, the CTV shifts can be employed as reference CTV shifts.

This study had four limitations. First, we should compare the reference CTV shifts with manual registration with those with implanted fiducial markers, because uncertainties with fiducial markers could be smaller than those in this study. Second, a small amount of patient data was used as the training datasets in this study. Therefore, the MLAs could be made more accurate with a higher generalization by increasing the patient data in the training datasets. Third, the anatomical features may not accurately reflect the rotation and deformation of CTVs, and thereby large errors might be caused as shown in Figs 5 and 6, and Table 5. It is necessary to consider the CTV rotation and deformation in future work. Finally, the anatomical features were extracted manually from the pCT and pretreatment CBCT images. Extraction of the anatomical features should be automated in the future. If the CTV shift predictions could be performed automatically by an automated feature extraction, a one-step image guided procedure would be feasible by combining the automated bone based positioning. As a result, automated CTV-based positioning based on anatomical features would be achieved.

In conclusion, this study developed a semi-automated prediction approach to CTV shifts using five types of MLAs with anatomical features between pCT and pretreatment CBCT images for improvement of the positioning PCa patients in IGRT. The results suggested that the MLAs with anatomical features could be useful in the prediction of CTV shifts. Furthermore, an anatomical feature associated with positional difference of the upper rectal wall, i.e., feature 3, could be used for prediction, and the prediction of CTV shifts was possible with small errors using only feature 3. In that case, only one click was required for feature selection, indicating that our proposed method could be a simple and effective one.

## CONFLICT OF INTEREST

None declared.
